# Research Progress on Risk Factors of Preoperative Anxiety in Children: A Scoping Review

**DOI:** 10.3390/ijerph19169828

**Published:** 2022-08-09

**Authors:** Weiwei Liu, Rui Xu, Ji’e Jia, Yilei Shen, Wenxian Li, Lulong Bo

**Affiliations:** 1 Faculty of Anesthesiology, Changhai Hospital, Naval Medical University, Shanghai 200433, China; 2 Department of Anesthesiology, Eye & ENT Hospital, Fudan University, Shanghai 200031, China

**Keywords:** children, preoperative anxiety, risk factors

## Abstract

Background: Preoperative anxiety has adverse effects on children and negative impacts on postoperative rehabilitation. Anesthesiologists can accurately identify children with preoperative anxiety, and individualized intervention can effectively improve their psychological state and clinical prognosis. However, a comprehensive summary of the current available evidence has yet to be conducted. Searches were conducted in Medline databases from inception to March 2022. Primary studies that reported preoperative anxiety in children and its attendant effects on postoperative recovery and prognosis were screened and included. Among the 309 publications identified, 12 related studies (*n* = 3540 patients) met the eligibility criteria. The incidence of preoperative anxiety in children in the included studies ranged from 41.7% to 75.44%. While 16 influencing factors were identified, only 5 factors had a significant impact on preoperative anxiety in children: younger age (*n* = 8), parental anxiety (*n* = 7), negative previous hospitalizations (*n* = 3), less sociableness (*n* = 2), and surgical setting (*n* = 1). The current scoping review identified risk factors for preoperative anxiety in children. Healthcare workers should identify and manage preoperatively anxious children. There are still some factors that are controversial, and large-scale clinical studies are needed.

## 1. Introduction

According to annual statistics released by the National Bureau of Statistics, the number of surgeries in China has increased annually [1]. With the increasing number of operations, the number of patients with preoperative anxiety also increases [2]. Children are particularly prone to preoperative anxiety due to their limited cognitive capabilities and greater dependence on others [3]. Several domestic and international studies have reported that 50%–80% of children experience preoperative anxiety [4,5,6,7]. Clinical manifestations of preoperative anxiety include fear, separation difficulties, and escape [8]. Approximately 25% of pediatric surgical procedures require physical bondage to complete the process of anesthesia induction, which increases the difficulty of anesthesia induction and the occurrence of postoperative agitation and delusion, and even leads to long-term behavioral abnormalities and psychological disorders in children [9]. Preoperative anxiety activates the human stress response system, leading to increased circulating glucocorticoid secretion, increased incidence of postoperative infection, and delayed wound healing, all of which have a negative impact on postoperative recovery in children [10]. Therefore, identifying and intervening in preoperative anxiety in children is an important link to improving clinical diagnosis and treatment.

This scoping review aims to systematically summarize current findings on the risk factors associated with preoperative anxiety in children, to help clinicians identify children with preoperative anxiety, and to provide targeted preoperative anxiety intervention measures, to provide a reliable theoretical and clinical basis for improving preoperative anxiety in children.

## 2. Methods

This scoping review is reported according to the PRISMA Extension for Scoping Reviews (PRISMA-ScR) Checklist [11]. We also followed the framework developed by Arksey and O’Malley for current review preparation [12]. In order to achieve the purposes of the scoping review, we employed a five-stage framework: (1) Determine the research question, (2) identify relevant published studies, (3) refine the study selection criteria, (4) collect relevant data from each included studies, and (5) summarize, report, and interpret the results.

The primary research question is what risk factors are associated with preoperative anxiety in children. We then performed a literature search in the electronic databases of Medline. We only included articles published in English without any year restriction. The Medline search strategy is “(((“Child”[Mesh] OR Children)) OR (“Pediatrics”[Mesh]) AND ((“Preoperative Period”[Mesh]) OR (Period, Preoperative))) AND ((“Anxiety”[Mesh]) OR (Angst) OR (Social Anxiety) OR (Anxieties, Social) OR (Anxiety, Social) OR (Social Anxieties) OR (Hypervigilance) OR (Nervousness) OR (Anxiousness))”. Reference lists of relevant systematic reviews were also searched to identify additional relevant studies. Articles were screened for eligibility by title and abstract by two reviewers (W.L., R.X.). The full texts of all qualified studies were reviewed by W.L. and R.X., and disputes were resolved through discussion (L.B.). The final list of the included studies was also reviewed by the authors for both completeness and relevance. A data extraction form was created and piloted by the research team. Detailed information of the included studies was collected, and factors related to preoperative anxiety in children were classified and synthesized by using both a numerical summary outlining the characteristics of the included studies and a narrative interpreting the results.

## 3. Results

**Figure 1:**
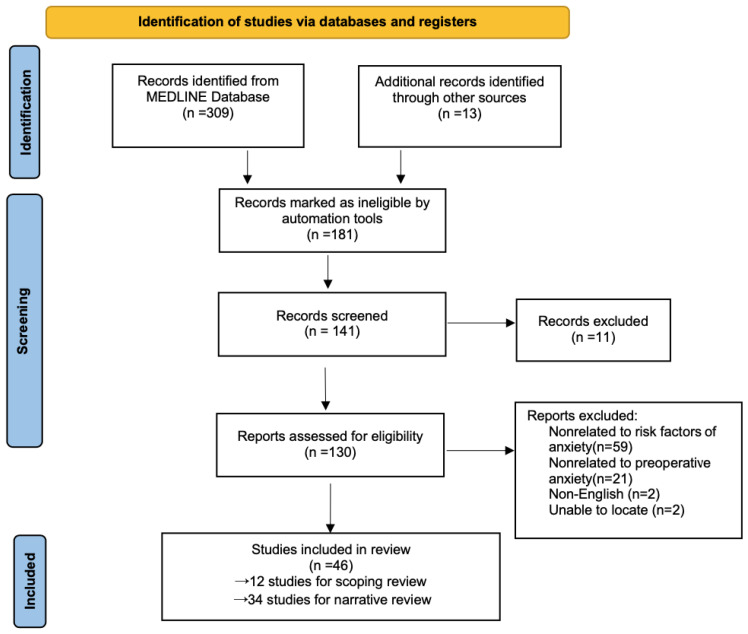
Flow of publication selection processes.

The literature search yielded a total of 309 references, with another 12 references identified through other sources. After screening, 216 references were excluded. A total of 105 references met the inclusion criteria (Figure 1). As shown in Table 1, a total of 12 included studies, with a total of 3540 patients, described the risk factors of preoperative anxiety in children, and thus were summarized. The incidence of preoperative anxiety in children in the included studies ranged from 41.7% to 75.44%. While 16 influencing factors were identified, only 5 factors had a significant impact on preoperative anxiety in children: younger age (*n* = 8) [7,13,14,15,16,17,18,19], parental anxiety (*n* = 7) [15,16,17,19,20,21,22], negative previous hospitalizations (*n* = 3) [15,16,20], less sociableness (*n* = 2) [18,21], and surgical setting (*n* = 1) [15] (Table 1). Meanwhile, the remaining literature was narratively interpreted.

### 3.1. Children’s Factors

#### 3.1.1. Age Factor

There is a close correlation between preoperative anxiety and age, and several studies have shown that the incidence of preoperative anxiety in younger children is much higher than in older children [7,13,14,15,16,17,18,19]. Getahun et al. studied 173 children aged 2–12 who underwent elective surgery, and the probability of anxiety in the operating room of children aged 2–6 was 3.83 times higher than that among children aged 7–12 [15].

The manifestation of preoperative anxiety also differs among the different age groups. Younger children (under 6 years old) mainly show separation anxiety, while older children often show fear of the pain caused by surgery, as well as anxiety caused by their own illness, disability, and inferiority complex [23]. Separation anxiety occurs in children aged 7 to 8 months and peaks in children as young as 1 year old [24,25]. In children younger than 7 months, there is often no separation anxiety when separated from their parents; a soothing voice, hug, and other physical actions can be used to comfort children and control the fasting time to the shortest time to facilitate smooth induction. However, in children aged between 7 months and 3 years, severe separation anxiety may occur, and drugs may be needed to reduce preoperative anxiety. Preoperative anxiety in children aged 3–6 years exists mainly in the fear of the surgery itself. Coping measures include preoperative education before surgery and during anesthesia. Other nonpharmacological means, such as game interactions and musical therapy, have been reported to play significant roles at this stage. In children aged 7–12 years, the response at this stage includes a more effective involvement in the preoperative preparation and induction of anesthesia. Effective preoperative mission, watching the video of popular science popularization, publicity, and explanation have been helpful. Additionally, some have found that having children hold a mask during induction of anesthesia may also be an effective intervention [26].

#### 3.1.2. Temperament Factor

There is a clear correlation between preoperative anxiety and the temperament of the child. Temperament is a stable psychological characteristic expressed in the intensity, speed, flexibility, and directivity of psychological activity [27]. Research shows that children with poor social skills are more likely to have preoperative anxiety [18,21]. Cheryl et al. conducted a meta-analysis of 12 related studies, comprising 1064 patients, and found that factors such as daily activities, emotional state, social situation, introversion, escape behavior, and degree of irritability were all correlated with the occurrence of preoperative anxiety in children. The analysis results revealed that the incidence of preoperative anxiety increased when children had daily negative emotions, irritability, introversion, and avoidance behaviors, while socially active children generally had a lower incidence of preoperative anxiety [28].

Reports have shown that the incidence of preoperative anxiety significantly increases in children with psychological problems before surgical procedures and anesthesia induction, and children with psychological problems are more likely to experience anxiety in a new environment or stress environment, such as an unfamiliar operating room or preoperative waiting room. The rate of preoperative anxiety in children with previous problems of anxiety, depression, and autism had increased according to a previous report [29]. Some children, such as those with mental problems and developmental or behavioral disorders, often need drug sedation and restraint measures to obtain their cooperation [30].

#### 3.1.3. Surgical Diagnosis and Treatment History Factors

Previous history of diagnosis requiring surgical interventions is closely related to preoperative anxiety in children. A review of 204 children aged 2–12 years who underwent elective surgery in Chile showed that previous negative experience with surgical procedures was a clear risk factor for preoperative anxiety [20]. Getahun et al. showed that children with a previous history of surgical anesthesia had a 5.96 times higher incidence of preoperative anxiety in the operating room than children with no history of surgical anesthesia [15].

#### 3.1.4. Other Factors

Other child-related factors include language barriers, low body weight, being the only child, and living in rural areas. The incidence of preoperative anxiety may be higher in such children [16,18,20,31].

A study by Mamtora et al. showed that children from Hispanic–Latino families had higher preoperative anxiety levels compared with native English speakers in the United States. However, in a study conducted in a Spanish-speaking country, the incidence of preoperative anxiety in Hispanic native children was lower than the rate observed in Hispanic–Latino children studied in the United States [16,20]. Owing to the complexity of medical terminologies, language barriers can increase communication difficulties, preventing child from understanding the diagnosis and surgical process, thus increasing the level of preoperative anxiety.

Chen et al. found that low body weight is an independent risk factor for preoperative anxiety in children [31]. Weight is an indicator of a child’s nutritional and physical conditions, and low body weight is associated with multiple factors. Shafina et al. demonstrated that parents’ educational background is related to their child’s body weight [32]. Well-nourished children may receive better family support and parental care in their daily lives, thus reducing their anxiety about facing unfamiliar environments.

The results of a Greek study of 128 children undergoing surgery and a Chinese study of 183 children undergoing eye surgery showed a higher incidence of preoperative anxiety in children without siblings [16,31]. The results also demonstrated that children without siblings may be more prone to anxiety than children with siblings [23]. Charana et al. reported higher anxiety among families living in rural areas than among urban residents [16].

**Table 1:** Summary of risk factors of preoperative anxiety in children.

First Author, Year	Type of Surgery, Participants	Type of Study	Age Group, Incidence of Preoperative Anxiety	Risk Factors
Liang Y, 2021 [7]	Elective surgery, 220	Cross-sectional survey study	2 to 7, 67.6%	Unschooled children, medical staff’s attention, the degree of cooperation when puncturing the venous needle
Chen A, 2021 [31]	Ophthalmic surgery, 183	Retrospective analysis	3 to 7, not described	Being the only child, lower body weight, parental educational level
Getahun AB, 2020 [15]	Elective surgery, 173	Cross-sectional observational study	2 to 12, 75.44%	Younger age, previous surgery and anesthesia, surgical setting, parental anxiety
Arze S, 2020 [20]	Elective outpatient or inpatient surgery, 204	Cohort study	2 to 12, 41.7%	Language barrier, parental anxiety, previous negative surgical experience
Charana A, 2018 [16]	Elective surgery, 128	Observational study	1 to 14, not described	High parental anxiety, age, being the only child, living in rural areas, education level, previous hospitalization
Mamtora PH, 2018 [18]	Outpatient adenoidectomy and/or tonsillectomy surgeries, 294	Cohort study	2 to 15, not described	Younger, less sociable children, language barriers
Malik R, 2018 [22]	Elective surgery, 60	Prospective observational study	7 to 12, 48%	Parental anxiety, socioeconomic background
Moura LA, 2016 [13]	Outpatient surgery, 210	Cross-sectional survey study	5 to 12, 42%	Age, socioeconomic status
Cui X, 2016 [17]	(ENT) plastic or ophthalmological surgeries, 102	Observational study	2 to 12, not described	Preschool children, parental anxiety
Kim JE, 2012 [14]	Elective surgery, 455	Prospective, observational study	2 to 12, 52.1%	Young age, long waiting times
Fortier MA, 2010 [21]	Outpatient tonsillectomy and adenoidectomy, 261	Prospective observational study	2 to 12, not described	Low child sociability, high parent anxiety
Davidson AJ, 2006 [19]	Any general anesthesia surgery, 1250	Prospective cohort study	3 to 12, 50.2%	healthcare attendances, longer duration of procedure, having more

### 3.2. Parental Factors

Multiple studies have shown that parental anxiety is associated with children’s preoperative anxiety [15,16,17,19,20,21,22]. In 2018, a study from the General Hospital of Alexandropoulos University in Greece showed that parental anxiety could negatively affect children [16]. A study by Getahun et al. found that children with anxious parents had a 3.43 times higher incidence of anxiety in the operating room than children with nonanxious parents [15]. Similarly, studies have shown that parental anxiety in the preoperative waiting room is positively correlated with perioperative anxiety in children [20]. Preoperative anxiety in younger children was more significantly associated with parental anxiety, as preschoolers were more attached to their parents [17].

### 3.3. Surgical Factors

#### 3.3.1. Environmental Factors

The environment of the operating room is also an important factor affecting preoperative anxiety in children. The brightness of the lighting in the operating room, the alarm sound of the instrument, and the noise from the surgical team can significantly increase the anxiety level of children [33]. A quiet and comfortable environment with music and the use of special guidance methods can significantly reduce the preoperative anxiety of children, thus increasing their compliance [34].

#### 3.3.2. Operation Type

Reports on the effects of outpatient and inpatient surgical procedures on preoperative anxiety in children are inconsistent. Getahun et al. showed that the incidence of operating theater anxiety was 5.67 times higher in outpatient children than in hospitalized children [15]. However, Davidson et al. observed that surgical procedures on outpatient children resulted in lower preoperative anxiety than hospitalized patients [19].

A study on whether the introduction of pediatric anesthesia cartoon information leaflets could reduce the level of preoperative anxiety in children undergoing major surgery found that the preoperative anxiety level of children undergoing neurosurgery (median of 33 points) was higher than that of children undergoing orthopedic and abdominal surgery (median of 30 points) [4].

#### 3.3.3. Preoperative Waiting Area Environment and Waiting Time

The environment of the preoperative waiting area and the waiting time may have an impact on the preoperative anxiety in children. Scarano et al. showed that being relatively familiar with the environment of the preoperative waiting area, or performing activities in a room with toys, can effectively reduce preoperative anxiety in children [35]. Prolonged waiting time in the preoperative waiting area increases anxiety, and some scholars have reported that the time in the preoperative waiting area exceeding 10 min will significantly increase the need for sedation [14].

### 3.4. Anesthesia Factors

In the perioperative period, children have the greatest anxiety during anesthesia induction [7,22,36]. The influence of different anesthesia induction methods (intravenous or inhalational induction) on anxiety in children remains controversial. Some studies have reported that the main cause of preoperative anxiety in children during intravenous induction comes from venous catheterization [37]. An unpleasant preoperative puncture experience increases the risk of preoperative anxiety in children [7]. Therefore, during preoperative venipuncture, the medical and nursing staff can reduce the pain of puncture and reduce children’s inner fear by using encouraging words, preoperative education, or the use of local anesthetics. Inhalation induction also increases anxiety in children, mainly because of the fear of the face mask and the pungent smell generated with the increasing concentration of volatile anesthetic drugs [38]. The study revealed that parent-guided mask exposure exercises on the day of surgery could increase compliance [39].

### 3.5. Other Factors

Other factors that increase preoperative anxiety in children include long hospital stays, lack of anesthesia and surgical safety knowledge, and attitudes of nurses and physicians [19,40,41]. The length of stay may indicate the severity and extent of the surgical procedure and may also reflect increased anxiety due to separation from the child’s usual environment [42]. Shaheen et al. showed that age-appropriate anesthetic and surgical information can reduce preoperative anxiety and enhance cooperation [40]. Furthermore, sympathetic attitudes of nurses and physicians can reduce the level of preoperative anxiety in children [41].

## 4. Discussion

Overall, our scoping review explores the risk factors of preoperative anxiety in children. Younger age, parental anxiety, negative previous hospitalizations, less sociableness, and surgical setting were identified as risk factors, and may play a role in terms of perioperative management of pediatric patients undergoing surgical procedures. Given the small amount of relevant literature included for the scoping review, we also narratively reviewed the remaining literature and discussed how the children, parent, surgery, and anesthesia were involved in preoperative anxiety in this population.

Anxiety is an adverse emotion that usually refers to uneasiness about an upcoming event or uncertain outcome. In general, preoperative anxiety is usually caused by a perceived lack of control [43]. Severe anxiety symptoms can affect the overall condition of the patient, leading to a range of complications. The perception of anxiety is closely related to the child’s developmental stage and cognitive ability, which is positively correlated with the age of the child. With increasing age, children’s cognitive ability improves significantly, and their ability to resist pressure also increases [44]. Thus, the incidence of preoperative anxiety is higher in younger children and decreases with increasing age. Children with poor social skills are usually considered to be introverted and seem to be more rigid and inflexible in new or stressful environments [45]. This may make them more anxious in unfamiliar and stressful environments, such as the surgical environment.

Children’s previous negative experience of surgery and anesthesia also has a negative impact on their behavior in subsequent anesthesia and surgery [19,21]. Studies have found that to strengthen the attention and care of children, preoperative use of sedative drugs, such as midazolam and dexmedetomidine, to provide effective postoperative analgesia, helps cultivate a close relationship between children and surgeons and anesthesiologists [46]. Therefore, to ensure smooth implementation of the diagnosis, treatment, and surgical procedure, reducing postoperative complications can greatly reduce the incidence of preoperative anxiety in subsequent surgical procedures.

Parents have an important influence on children’s emotional response to surgical stress [47]. The possible mechanisms related to the influence of parents’ emotional factors on children’s preoperative anxiety include common genetic, social, and environmental factors. When dealing with unfamiliar stress, children are often influenced by parents’ advice and guidance. Parents’ anxiety about the risks related to anesthesia and surgical procedures may resonate with children. Parents’ effective management of personal anxiety can greatly reduce the incidence of preoperative anxiety of their children and improve the results of surgical intervention and the emotional state on the day of surgery [48]. Therefore, reducing the preoperative anxiety of parents is very important to improve the preoperative anxiety of children [49]. Parental characteristics that affect their anxiety level include the age of children and parents, female gender, high and low education, living in rural areas, and their anxiety as a characteristic. Identifying these characteristics can help develop and implement appropriate anxiety control interventions [16].

The anxiety of children in the induction period mainly comes from separation from their parents, the operating room environment, and the presence of masked individuals interacting with the children [50]. Children exposed to low-level sensory stimuli during the induction of anesthesia and those who are exposed to background music exhibit lower levels of anxiety and increased compliance [34]. Other reporting factors include language barrier, being the only child, living in rural areas, parental education level, lower body weight, behavioral problems with previous healthcare attendances, longer duration of procedure, having more than five previous hospital admissions, and socioeconomic status. There are still some controversies about the influence of these factors on preoperative anxiety.

Our review should be interpreted in light of several limitations. First, we restricted our literature search within the Medline database and with a language restriction to English; nevertheless, we may have missed potential literature in other databases and languages. Second, our outcome of interest was preoperative anxiety, and the intervention measures were not reviewed systematically, which deserves further research. Lastly, we did not conduct a formal quality assessment of included studies, which might lead to potential biases. A future systematic review should provide corresponding intervention measures for different causes of preoperative anxiety.

## 5. Conclusions

Preoperative anxiety affects millions of pediatric patients each year and may have both short- and long-term adverse effects after surgery [51]. Clarifying the factors affecting preoperative anxiety in children has an important role to play in reducing preoperative anxiety. The current scoping review identified risk factors for preoperative anxiety in children, including young age, negative medical experience, parents with high anxiety, operating room environment, and children with poor social skills. Other factors, such as being the only child, parents’ education level, language environment, operation type, anesthesia induction method, still need large-scale clinical research to predict children’s preoperative anxiety. Taken together, our findings provide a synthesis of risk factors of preoperative anxiety in children and highlight specific areas and topics requiring further studies. Healthcare workers are urged to develop personalized prevention or intervention strategies, reduce the use of opioid drugs, and rationally allocate medical resources to minimize the occurrence of preoperative anxiety in children.

## Data Availability

Not applicable.

## References

[1] National Bureau of Statistics of China Number Of Surgeries in Chinese Healthcare Institutions 2018. http://www.stats.gov.cn/english/.

[2] Li X.R., Zhang W.H., Williams J.P., Li T., Yuan J.H., Du Y., Liu J.D., Wu Z., Xiao Z.Y., Zhang R. (2021). A multicenter survey of perioperative anxiety in China: Pre- and postoperative associations. J. Psychosom. Res..

[3] Lee J.H., Jung H.K., Lee G.G., Kim H.Y., Park S.G., Woo S.C. (2013). Effect of behavioral intervention using smartphone application for preoperative anxiety in pediatric patients. Korean J. Anesthesiol..

[4] Kassai B., Rabilloud M., Dantony E., Grousson S., Revol O., Malik S., Ginhoux T., Touil N., Chassard D., Neto E.P.D.S. (2016). Introduction of a paediatric anaesthesia comic information leaflet reduced preoperative anxiety in children. Br. J. Anaesth..

[5] Perry J.N., Hooper V.D., Masiongale J. (2012). Reduction of Preoperative Anxiety in Pediatric Surgery Patients Using Age-Appropriate Teaching Interventions. J. Perianesth. Nurs..

[6] Chow C.H., Van Lieshout R.J., Schmidt L.A., Dobson K.G., Buckley N. (2016). Systematic Review: Audiovisual Interventions for Reducing Preoperative Anxiety in Children Undergoing Elective Surgery. J. Pediatr. Psychol..

[7] Liang Y., Huang W., Hu X., Jiang M., Liu T., Yue H., Li X. (2021). Preoperative anxiety in children aged 2–7 years old: A cross-sectional analysis of the associated risk factors. Transl. Pediatr..

[8] Yang Y., Zhang M., Sun Y., Peng Z., Zheng X., Zheng J. (2022). Effects of advance exposure to an animated surgery-related picture book on preoperative anxiety and anesthesia induction in preschool children: A randomized controlled trial. BMC Pediatr..

[9] Kain Z.N., Wang S.M., Mayes L.C., Caramico L.A., Hofstadter M.B. (1999). Distress During the Induction of Anesthesia and Postoperative Behavioral Outcomes. Anesthesia Analg..

[10] Kain Z.N., Sevarino F., Alexander G.M., Pincus S., Mayes L.C. (2000). Preoperative anxiety and postoperative pain in women undergoing hysterectomy. A repeated-measures design. J. Psychosom. Res..

[11] Tricco A.C., Lillie E., Zarin W., O’Brien K.K., Colquhoun H., Levac D., Moher D., Peters M.D.J., Horsley T., Weeks L. (2018). PRISMA Extension for Scoping Reviews (PRISMA-ScR): Checklist and explanation. Ann. Intern. Med..

[12] Arksey H., O’Malley L. (2005). Scoping studies: Towards a methodological framework. Int. J. Soc. Res. Methodol..

[13] De Moura L.A., Dias I.M., Pereira L.V. (2016). Prevalence and factors associated with preoperative anxiety in children aged 5-12 years 1. Rev. Lat.-Am. Enferm..

[14] Kim J., Jo B., Oh H., Choi H., Lee Y. (2012). High anxiety, young age and long waits increase the need for preoperative sedatives in children. J. Int. Med. Res..

[15] Getahun A.B., Endalew N.S., Mersha A.T., Admass B.A. (2020). Magnitude and Factors Associated with Preoperative Anxiety Among Pediatric Patients: Cross-Sectional Study. Pediatr. Health Med. Ther..

[16] Charana A., Tripsianis G., Matziou V., Vaos G., Iatrou C., Chloropoulou P. (2018). Preoperative Anxiety in Greek Children and Their Parents When Presenting for Routine Surgery. Anesthesiol Res. Pract..

[17] Cui X., Zhu B., Zhao J., Huang Y., Luo A., Wei J. (2016). Parental state anxiety correlates with preoperative anxiety in Chinese preschool children. J. Paediatr. Child Health.

[18] Mamtora P.H., Kain Z.N., Stevenson R.S., Golianu B., Zuk J., Gold J.I., Fortier M.A. (2018). An evaluation of preoperative anxiety in Spanish-speaking and Latino children in the United States. Pediatr. Anesth..

[19] Davidson A.J., Shrivastava P.P., Jamsen K., Huang G.H., Czarnecki C., Gibson M.A., Stewart S.A., Stargatt R. (2006). Risk factors for anxiety at induction of anesthesia in children: A prospective cohort study. Pediatr. Anesth..

[20] Arze S., Lagos C., Ibacache M., Zamora M., González A. (2020). Incidence and risk factors of preoperative anxiety in Spanish-speaking children living in a Spanish-speaking country. Pediatr. Anesth..

[21] Fortier M.A., Del Rosario A.M., Martin S.R., Kain Z.N. (2010). Perioperative anxiety in children. Paediatr. Anaesth..

[22] Malik R., Yaddanpudi S., Panda N.B., Kohli A., Mathew P.J. (2018). Predictors of Pre-operative Anxiety in Indian Children. Indian J. Pediatr..

[23] Quiles Sebastián M.F., Méndez Carrillo F., Ortigosa Quiles J. (2001). Preocupaciones prequirúrgicas: Estudio empírico con población infantil y adolescente [Pre-surgical worries: An empirical study in the child and adolescent population]. An. Esp. Pediatr..

[24] Kain Z.N., Caldwell-Andrews A.A., LoDolce M.E., Krivutza D.M., Wang S.M. (2002). The peri operative behavioral stress response in children. Anesthesiology.

[25] Ahmed M.I., Farrell M.A., Parrish K., Karla A. (2011). Preoperative anxiety in children risk factors and non-pharmacological management. Middle East J. Anaesthesiol..

[26] Dave N.M. (2019). Premedication and induction of anaesthesia in paediatric patients. Indian J. Anaesth..

[27] Goldsmith H.H., Lemery K.S., Aksan N., Buss K.A., Molfese V.J., Molfese D.L. (2000). Temperament substrates of personality development. Temperament and Personality Development across the Life-Span.

[28] Chow C.H.T., Rizwan A., Xu R., Poulin L., Bhardwaj V., Van Lieshout R.J., Buckley N., Schmidt L.A. (2019). Association of Temperament With Preoperative Anxiety in Pediatric Patients Undergoing Surgery: A Systematic Review and Meta-analysis. JAMA Netw. Open.

[29] Fortier M.A., Martin S.R., Chorney J.M., Mayes L.C., Kain Z.N. (2011). Preoperative anxiety in adolescents undergoing surgery: A pilot study. Pediatr. Anesth..

[30] Campbell R.L., Shetty N.S., Shetty K.S., Pope H.L., Campbell J.R. (2018). Pediatric Dental Surgery Under General Anesthesia: Uncooperative Children. Anesthesia Prog..

[31] Chen A., Sheng H., Xie Z., Shen W., Chen Q., Lin Y., Gan X. (2021). Prediction of preoperative anxiety in preschool children undergoing ophthalmic surgery based on family characteristics. J. Clin. Anesth..

[32] Noor Shafina M.N., Abdul Rasyid A., Anis Siham Z.A., Nor Izwah M.K., Jamaluddin M. (2020). Parental perception of children’s weight status and sociodemographic factors associated with childhood obesity. Med. J. Malaysia..

[33] Utrillas-Compaired A., De la Torre-Escuredo B.J., Tebar-Martínez A.J., Barco A.-D. (2014). Does preoperative psychologic distress influence pain, function, and quality of life after TKA?. Clin. Orthop. Relat. Res..

[34] Kain Z.N., Wang S.M., Mayes L.C., Krivutza D.M., Teague B.A. (2001). Sensory stimuli and anxiety in children undergoing surgery: A randomized, controlled trial. Anesth. Analg..

[35] Scarano F., Corte A., Michielon R., Gava A., Midrio P. (2021). Application of a non-pharmacological technique in addition to the pharmacological protocol for the management of children’s preoperative anxiety: A 10 years’ experience. La Pediatr. Med. Chir..

[36] Chorney J.M., Kain Z.N. (2009). Behavioral Analysis of Children’s Response to Induction of Anesthesia. Anesth. Analg..

[37] Perrott C., Lee C.-A., Griffiths S., Sury M.R.J. (2017). Perioperative experiences of anesthesia reported by children and parents. Pediatr. Anesth..

[38] Przybylo H., Tarbell S., Stevenson G. (2005). Mask fear in children presenting for anesthesia: Aversion, phobia, or both?. Pediatr. Anesth..

[39] Walker K.L., Wright K.D., Raazi M. (2018). Randomized-controlled trial of parent-led exposure to anesthetic mask to prevent child preoperative anxiety. Étude randomisée contrôlée d’une exposition des enfants au masque anesthésique par un parent pour prévenir l’anxiété préopératoire des jeunes patients. Can. J. Anaesth..

[40] Shaheen A., Nassar O., Khalaf I., Kridli S.A., Jarrah S., Halasa S. (2018). The effectiveness of age-appropriate pre-operative information session on the anxiety level of school-age children undergoing elective surgery in Jordan. Int. J. Nurs. Pract..

[41] Tabrizi J.S., Seyedhejazi M., Fakhari A., Ghadimi F., Hamidi M., Taghizadieh N. (2015). Preoperative Education and Decreasing Preoperative Anxiety Among Children Aged 8–10 Years Old and Their Mothers. Anesthesiol. Pain Med..

[42] Davidson A., Howard K., Browne W., Habre W., Lopez U. (2011). Preoperative Evaluation and Preparation, Anxiety, Awareness, and Behavior Change. Gregory’s Pediatric Anesthesia.

[43] Watson A.T., Visram A. (2003). Children’s preoperative anxiety and postoperative behaviour. Pediatr. Anesth..

[44] Muris P., Mayer B., Freher N.K., Duncan S., Hout A.V.D. (2010). Children’s internal attributions of anxiety-related physical symptoms: Age-related patterns and the role of cognitive development and anxiety sensitivity. Child. Psychiatry Hum. Dev..

[45] Eisenberg N., Spinrad T.L., Fabes R.A., Reiser M., Cumberland A., Shepard S.A., Valiente C., Losoya S.H., Guthrie I.K., Thompson M. (2004). The relations of effortful control and impulsivity to children’s resiliency and adjustment. Child. Dev..

[46] Kayaalp L., Bozkurt P., Odabasi G., Dogangun B., Cavusoglu P., Bolat N., Bakan M. (2004). Psychological effects of repeated general anesthesia in children. Pediatr. Anesth..

[47] Li H.C., Lopez V., Lee T.L. (2007). Psychoeducational preparation of children for surgery: The importance of parental involvement. Patient Educ. Couns..

[48] Creswell C., Waite P. (2015). The Dynamic Influence of Genes and Environment in the Intergenerational Transmission of Anxiety. Am. J. Psychiatry.

[49] Santapuram P., Stone A.L., Walden R.L., Alexander L. (2021). Interventions for Parental Anxiety in Preparation for Pediatric Surgery: A Narrative Review. Children.

[50] Kain Z., Mayes L., Borestein M., Genevro J. (1996). Anxiety in children during the perioperative period. Child Development and Behavioral Pediatrics.

[51] Fronk E., Billick S.B. (2020). Pre-operative Anxiety in Pediatric Surgery Patients: Multiple Case Study Analysis with Literature Review. Psychiatr. Q..

